# “Heroes” and “Villains” of World History across Cultures

**DOI:** 10.1371/journal.pone.0115641

**Published:** 2015-02-04

**Authors:** Katja Hanke, James H. Liu, Chris G. Sibley, Dario Paez, Stanley O. Gaines, Gail Moloney, Chan-Hoong Leong, Wolfgang Wagner, Laurent Licata, Olivier Klein, Ilya Garber, Gisela Böhm, Denis J. Hilton, Velichko Valchev, Sammyh S. Khan, Rosa Cabecinhas

**Affiliations:** 1 Jacobs University Bremen, Bremen International Graduate School of Social Sciences, Bremen, Germany; 2 Victoria University of Wellington, School of Psychology, Wellington, New Zealand; 3 University of Auckland, School of Psychology, Auckland, New Zealand; 4 University of the Basque Country, School of Psychology, San Sebastián, Spain; 5 Brunel University, School of Social Sciences, London, United Kingdom; 6 Southern Cross University, Department of Psychology, Lismore, Australia; 7 National Singapore University, Institute of Policy Studies, Singapore, Singapore; 8 Johannes Kepler University, Department of Social and Economic Psychology, Linz, Austria; 9 Université Libre de Bruxelles, Center for Social and Cultural Psychology, Bruxelles, Belgium; 10 Saratov State University, Department of Pedagogy and Psychology of Professional Education, Saratov, Russia; 11 University of Bergen, Faculty of Psychology, Bergen, Norway; 12 University of Toulouse II, Cognition, Langues, Langage et Ergonomie, Toulouse, France; 13 Tilburg University, Department of Cross-Cultural Psychology, Tilburg, Netherlands; 14 University of Exeter, Medical School, Exeter, United Kingdom; 15 University of Minho, Institute of Social Sciences, Braga, Portugal; MIT, UNITED STATES

## Abstract

Emergent properties of global political culture were examined using data from the World History Survey (WHS) involving 6,902 university students in 37 countries evaluating 40 figures from world history. Multidimensional scaling and factor analysis techniques found only limited forms of universality in evaluations across Western, Catholic/Orthodox, Muslim, and Asian country clusters. The highest consensus across cultures involved scientific innovators, with Einstein having the most positive evaluation overall. Peaceful humanitarians like Mother Theresa and Gandhi followed. There was much less cross-cultural consistency in the evaluation of negative figures, led by Hitler, Osama bin Laden, and Saddam Hussein. After more traditional empirical methods (e.g., factor analysis) failed to identify meaningful cross-cultural patterns, Latent Profile Analysis (LPA) was used to identify four global representational profiles: Secular and Religious Idealists were overwhelmingly prevalent in Christian countries, and Political Realists were common in Muslim and Asian countries. We discuss possible consequences and interpretations of these different representational profiles.

## Introduction

As processes of globalization connect the world [1], an important question arises: what might be the basis of any possible global political culture? If such a confluence is likely, cultures with different historical trajectories and political traditions will need to find ways to work together not only economically, but also politically. In this evolving framework of globalization, tradition, according to political theorists following Edmund Burke [2] provides a “general bank and capital of nations and of ages” (p. 144) that is a superior guarantor of social order and societal well-being compared to abstractions like “liberty” and “the rights of man” as emphasized in the French Revolution. How history is represented is an important warrant of legitimacy, or “charter” for global political order, and the emergence of a diverse but interconnected world political culture [3], [4]. Through the embellishment of history into tradition [5], historical figures perceived and evaluated as either positive or negative become embodiments of national political cultures that may collude or collide against one another. This paper aims to shed some light on how historical figures are perceived and evaluated and whether there are consistencies or inconsistencies across 37 countries.

Acquiring representations of major historical figures is a principal mechanism through which political socialization occurs. For example, some of the first robust political knowledge acquired by a child is beliefs about the character of the chief executive of their land [6], [7]. American children learn about “Honest Abe Lincoln” and “George Washington not telling a lie about chopping down the cherry tree” [8], [9] as morality tales through which virtues suitable for participation in liberal democracy are communicated [10]. Chinese children may learn instead about the strength Mao Zedong demonstrated swimming the Yangtze River in his 70s (signaling the start of the cultural revolution) and the Qin Emperor escaping assassination to unify China (popularized in movies like Zhang Yimou’s *Hero*) [11]. These contrasting morality tales about the invincibility of central authority accord with variances in power distance [12] across national political cultures and they make the point that people’s interpretations and evaluations of who is a “hero” and who is a “villain” in world history differ across countries. However, in the absence of large-scale cross-cultural surveys of figures in world history, the question of the existence of shared understandings of “heroes” and “villains” across countries remains open. An equivalent understanding of these figures could help us begin to understand some components of what might constitute "global political culture”. The outcome of this issue connects to lively debates across the social sciences about the nature, extent and universality of the cosmopolitanism emerging from globalization [13], [14].

Some culture theory in anthropology [15] and major cross-cultural theories in psychology [12], [16] concur that the world can be carved up into regions or cultural zones where different modal understandings are likely to emerge. However, anthropologists have also focused on processes by which the boundaries between countries have become more fluid, allowing people to have more contact with each other and a “*cultural flow* of capital, people, commodities, images and ideologies through which the spaces of the globe are becoming increasingly intertwined” (p. 2) [17]. Authors such as Appadurai [18] have theorized that mass media and migration have created multiple landscapes of global cultural flows characterized by complexity, overlap, and disorder, where new forms of identity formation that are often de-territorialized emerge in reaction to dominant Western influences [18], [19]. This contrasts with the more orderly conception of globalization associated with cosmopolitan theorists such as Nussbaum [13], who sees no conflict between being a citizen of the world and local identifications, but rather argues for education that builds up broader and broader concentric circles of identification around the self to finally encompass humanity. Her conception is consistent with ideas about the global circulation of Western ideologies, composed of elements from an Enlightenment worldview valuing freedom, equality, democracy and rationality, which act as ideological formations that work to produce a cultural homogenization of the world [17], [20]. Such formations are highly controversial [14], [21].

Research on political culture [22] has, to-date, focused exclusively on the persistence of *democratic* cultures and regimes, which seems an imbalanced proposition for a century where, for example, the rise of non-democratic China has been so salient. Indeed, Fuchs [22] closed his chapter on political culture with a call for “broadening the scope of the paradigm to countries that have either autocratic regimes or are regimes in the democratization process” (p. 179). He further questioned whether “the influential bearers of the political culture in those [non-democratic] regimes are average citizens” (p. 179). The current research broadens the scope of our understanding of political culture in non-democratic or recently democratizing states by examining how some of the most important figures in world history are represented across cultures, including many non-Western cultures and non-democratic states. In so doing, we begin to form an understanding and to answer the question of whether and how political culture in these states is carried by ordinary (albeit educated) citizens.

Given that the survey methods employed here require literacy and some knowledge of world history, our investigation is necessarily restricted to people living with modernity, but goes beyond Western forms of modernity. The more extensive (and frequently cosmological) concepts of personhood examined by anthropologists like Fowler [23] are outside the scope of this investigation. We accept his position that “since personhood is heavily entangled with other factors of identity, there can be no single definition that applies to all contexts, nor any single process through which personhood is attained” (p. 156). The context examined here is underpinned by the contemporary state, through one of its key socializing institutions-education in particular—at secondary and higher levels.

In a related study [24], involving university students in 30 countries around the world, it was found that 40 of the most important events in world history were not evaluated in a universally shared way. Instead, “Historical Calamities”, “Historical Progress”, and “Historical Resistance to Oppression” were identified as specific latent factors (or concepts) carrying discrete forms of shared evaluative meaning about historical events across cultures. Every country had similar ideas about what events were regarded as calamitous (e.g., the World Wars, atomic bombings, global warming), while there was less evaluative agreement on progress (e.g., digital age, man on the moon, Industrial Revolution) and considerable disagreement about resistance to oppression (or Human Rights, e.g., American Civil War, abolition of slavery, Fall of the Berlin Wall). Multilevel analysis found that individuals who viewed “Historical Calamities” less negatively and “Historical Progress” more positively were more willing to fight for their country in a war. The size of these relationships varied across cultures, and the placement of these events on an overall multidimensional space of meaning also varied considerably according to the cultural zone of the people rating the events. Events that have radically transformed Western societies, such as women’s emancipation, did not fit into a stable position in a multidimensional space or contribute reliably to any cross-cultural scale measures because of disputation around their evaluative meaning in different cultural zones [24]. These findings suggest limited universality of cross-cultural meanings regarding important events in world history, even among a restricted sample of educated young people.

Historical figures, however, may serve a different function in national political cultures compared to events. Historical figures can symbolize, objectify [25] and embody national [8] and civilizational political cultures, whereas critical events like World War II are more like cultural schemata that may be invoked or mobilized as lessons to justify action [4], [26]. Events impart lessons, whereas “heroes” embody values and inspire actions [27]. Both must be interpreted through cultural frames: to paraphrase Marshall Sahlins, “Culture does not make history so much as make sense of it” [28]. In common language, it is easy to talk about Christian, Confucian, Buddhist, or Mohammedan civilizations, referring to founding historical figures in a way that is much more difficult for an event. This is because Jesus, Confucius, Buddha, and Mohammed have come to embody values and teach philosophies for living with greater salience than events [29], [30], [31]—they are interpreted to have exerted systematic agency on the course of human events [28].

Hence, our initial research questions are descriptive. Among 40 of the more important figures in world history (see Procedure and Material section for description of the selection of the historical figures), who are regarded as the most positive and the most negative? Are these evaluations consistent or inconsistent across cultures and congruent clusters of countries? Is there consistency in the types of figures evaluated as good and bad across cultures and such cultural zones? Answers to these questions will inform us of a potential emergent global political culture: If consensus exists as to the “heroes” and “villains” of world history, then these figures should symbolize the values and achievements that humanity aspires to and humanity, as a whole, rejects. Folk psychologies are rich in theories about people and may supply meaningful structure to evaluative ratings of historical figures: Scientists, presidents, dictators, and humanitarians are all familiar social categories that may be employed to shape person perception of historical figures [32], [33], [34].

Based on the results of our earlier research [24], we anticipate a lack of universality in the evaluation of major figures in world history. We expect that figures are likely to be the object of nationalistic social identification [26], [29]: An American is far more likely to identify with George Washington and Abraham Lincoln (and therefore rate them more positively) than Mao Zedong or Sun Yatsen, whereas the opposite pattern is likely to be true for people living in China. To the extent that national “heroes” (or “villains”) anchor the dimensions of variation for the evaluation of figures in world history, we might therefore expect different dimensional spaces to emerge in different parts of the world, suggesting regional variations in global political culture. However, given the prevalence of cultural interchange, and previous research showing the preeminence of Western influences in history around the globe [35], [36], an analytical strategy based on a priori country based differences was deemed inadequate. A new concept, namely, that of representational profiles [37], is introduced to measure emergent ensembles of meaning that may hold across cultures, but retain a commitment to group-based and plural structures of meaning.

### Historical Representational Profiles

We employ Latent profile analysis (LPA) to build a typology of the responses shared between individuals as a way to manage this heterogeneity and expected plurality of meanings. We term these classifications of patterns of individual differences in rating historical figures “representational profiles” [37].

We define a representational profile as a set of discretely measureable attitudes and beliefs bound together by a system of meaning that is used by people to make sense of a particular social object, such as figures in world history [28]. Each representational profile is a configuration of attitudes and beliefs that can be communicated in a meaningful way to other people who may or may not agree with this point of view. LPA maintains an ontological commitment to groups without requiring an a priori commitment to the representations being a property of pre-existing social categories, like nationality or ethnicity. We show how the two techniques can work hand in glove to capture the complexity of how people express their beliefs and views about historical figures in a worldwide data set.

## Method

### Participants

Data were collected initially from 6,902 university students who were citizens of 37 countries (Argentina, Australia, Austria, Belgium, Brazil, Bulgaria, Canada, China, Colombia, Fiji, France, Germany, Hong Kong, Hungary, India, Indonesia, Italy, Japan, Malaysia, Mauritius, Mexico, Netherlands, Norway, New Zealand, Pakistan, Peru, Philippines, Portugal, Russia, Singapore, South Korea, Spain, Switzerland, Taiwan, Tunisia, UK, and USA). Sample sizes ranged from 78 (Peru) to 346 (Argentina) with an average of 182; most were collected between 2007 and 2008, but the UK sample was collected in 2010. Social science students were preferred, and specialists majoring in history were avoided. Participants with more than 33% missing values were excluded from the overall analyses (175 cases) because they were deemed to have demonstrated insufficient knowledge/interest in historical figures. Additional missing data in the LPA was estimated using FIML. The final sample thus consisted of 6,727 university students (60.8% were female, 36.7% were male, and 2.5% did not indicate their gender). Participants’ age ranged from 16 to 80 years (*M* = 21.73, *SD* = 5.19), with country age means ranging from 19.00 (Philippines) to 34.09 (Pakistan). This sampling approach ensures that our findings and conclusions derive from a truly cross-cultural sample that is not WEIRD (ie. Western-Educated-Industrialized-Rich-Democratic) [38]. Yet, the sample is homogenous in terms of educational levels, enabling us to be confident about the comparability of our findings across cultures.

### Ethics statement

The study was approved by the Ethics Committee of Victoria University of Wellington (VUW), New Zealand (0479JUN; 5th of June 2007). All data collection took place in universities with predominately undergraduate students in accord with the ethical regulations of that institution. The research was always anonymous, an information sheet was supplied, verbal information was always issued, potential participants were able to ask questions before filling-out the survey and informed consent was always implied by voluntary participation as set out in sections 4.1, 4.5(d) and 4.8(g) of the Human Ethics Policy of VUW. Potential participants were provided with information regarding the content of the study, the length of the survey, the procedures for anonymizing responses and the option to withdraw from the study at any stage without having to give reason and without any negative consequences. Regarding the procedures for the anonymizing of responses, participants were informed that data recorded in the study would be aggregated so that individual persons would not be identifiable. Feedback was provided through a written debriefing according to regulations set out in section 7, Appendix 1 of the Human Ethics Policy of VUW. In countries in which there were no ethics committees, the New Zealand ethics regulations were followed. The age of the university students depended on the graduating age of the local schooling systems in the participating countries. Participants’ privacy, rights and welfare were protected throughout the entire research procedure.

### Procedure and Materials

The survey consisted of a set of 40 historical figures. The choice of 40 historical figures was based on open-ended nominations from 24 countries [35], [36] using the criterion that a figure had to be among the top 10 people nominated in two or more countries. This was augmented by the inclusion of religious founders, who were mainly excluded from the previous research because of a 1000 year time limit for nominations. All questionnaires were translated from their original language into the language prevalent in the countries of administration and back-translated to ensure correct translation. Instructions for the participants were as follows: “Below is a list of historical figures. Please rate the intensity of your positive or negative feelings about each person (on a scale of 1 to 7, with 1 = extremely negative, 4 = neutral, and 7 = extremely positive), and how important you think each person is (on a scale of 1 to 7, with 1 = not at all important, 4 = fairly important, and 7 = extremely important). Please rate all the people, even if you don’t know much about them.” After this instruction the list of historical figures followed.

## Results

If not otherwise mentioned, we used SPSS 18 for all statistical analyses. Table 1 shows all figures sorted by descending means across all samples. The means overall were calculated on the basis of the mean per country. By doing this we eliminated the influence of the sample size.

**Table 1:** Descending means (M) evaluations of figures, standard deviations (SD) and Intraclass correlation coefficients (ICC) across 37 nations.

Evaluations of Figures			
Figure	M	SD	ICC
1. Albert Einstein	6.11	1.07	0.06
2. Mother Theresa	6.07	1.17	0.08
3. Mahatma Gandhi	5.90	1.19	0.13
4. Martin Luther King	5.84	1.15	0.17
5. Isaac Newton	5.74	1.14	0.05
6. Jesus Christ	5.55	1.48	0.14
7. Nelson Mandela	5.48	1.30	0.09
8. Thomas Edison	5.39	1.25	0.11
9. Abraham Lincoln	5.18	1.21	0.19
10. Buddha	5.06	1.51	0.04
11. Princess Diana	5.04	1.32	0.08
12. Columbus	5.03	1.31	0.07
13. Martin Luther	5.02	1.31	0.07
14. Bill Gates	4.95	1.40	0.09
15. Pope John Paul II	4.83	1.46	0.18
16. Mohammed	4.83	1.45	0.14
17. Franklin D. Roosevelt	4.82	1.21	0.10
18. George Washington	4.79	1.27	0.11
19. Karl Marx	4.79	1.38	0.12
20. Confucius	4.75	1.27	0.11
21. Alexander the Great	4.74	1.22	0.07
22. J.F. Kennedy	4.73	1.17	0.07
23. Winston Churchill	4.62	1.16	0.09
24. Che Guevara	4.60	1.31	0.09
25. Margaret Thatcher	4.34	1.28	0.12
26. Charlemagne	4.31	1.04	0.10
27. Sun Yatsen	4.21	0.86	0.26
28. Gorbachev	4.19	1.16	0.04
29. Deng Xiaoping	4.10	0.92	0.18
30. Napoleon	4.07	1.40	0.07
31. The Qin Emperor	4.00	0.96	0.04
32. Saladin	3.97	1.02	0.08
33. Genghis Khan	3.81	1.21	0.14
34. Lenin	3.66	1.36	0.15
35. Mao	3.50	1.33	0.19
36. Stalin	2.84	1.30	0.21
37. George Bush Jr	2.58	1.46	0.13
38. Saddam Hussein	2.41	1.26	0.27
39. Osama bin Laden	2.17	1.29	0.25
40. Adolf Hitler	1.76	1.15	0.16

The 10 most positively evaluated historical figures across all countries were (in descending order): Albert Einstein, Mother Theresa, Mahatma Gandhi, Martin Luther King, Isaac Newton, Jesus Christ, Nelson Mandela, Thomas Edison, Abraham Lincoln and Buddha. These are scientists, humanitarians, and religious figures with one exception of a political leader (Abraham Lincoln). It seems reasonable to argue that maybe Abraham Lincoln was perceived as a humanitarian, since it is known that he abolished slavery in the United States. The five most negatively evaluated historical figures across all countries were (from the bottom): Adolf Hitler, Osama bin Laden, Saddam Hussein, George W. Bush and Joseph Stalin. These are 20^th^ and 21^st^ century figures known for their roles in dictatorships, terrorism, mass murder, and/or driving their countries into unjust wars. They were followed by Mao Zedong, Vladimir Lenin, Genghis Khan, Saladin and The Qin Emperor. The last two had evaluations close to the neutral point of 4.04 and so are not evaluated as “villains” by our samples. Clearly time has dimmed the ferocious reputation of Genghis Khan, whose rating also approached the midpoint.

Intraclass correlation coefficients (ICC) [39] were calculated for all 40 evaluations of figures (see Table 1). An ICC greater than. 05 indicates a higher proportion of consistency (or correlation) in variability accounted for at the between-country level relative to variability between individuals within a country whereas an ICC lower than. 05 indicates relatively more variability accounted for at the individual level than at the country level [39], [40]. As seen in Table 1, some variation was available to be accounted for at the country level for most ratings of figures. In other words, the higher the ICC, the greater the cross-cultural compared to individual differences. The highest ICCs were for Saddam Hussein (0.27), Osama bin Laden (0.25) and Sun Yatsen (0.26), all figures who were evaluated more positively in one cultural zone compared to all others (Osama bin Laden and Saddam Hussein higher in Muslim, Sun Yatsen higher in Chinese societies). The lowest ICCs in terms of evaluations were for the Qin Emperor (0.04) who was rated as neutral in all countries, and Isaac Newton (0.05) and Albert Einstein (0.06) who were positively evaluated in all countries. This reflects an underlying agreement across all countries that Albert Einstein is a positive figure whereas Osama bin Laden draws different evaluations from Muslim and non-Muslim countries. These and other additional analyses are available from the authors on request.

**Fig 1:**
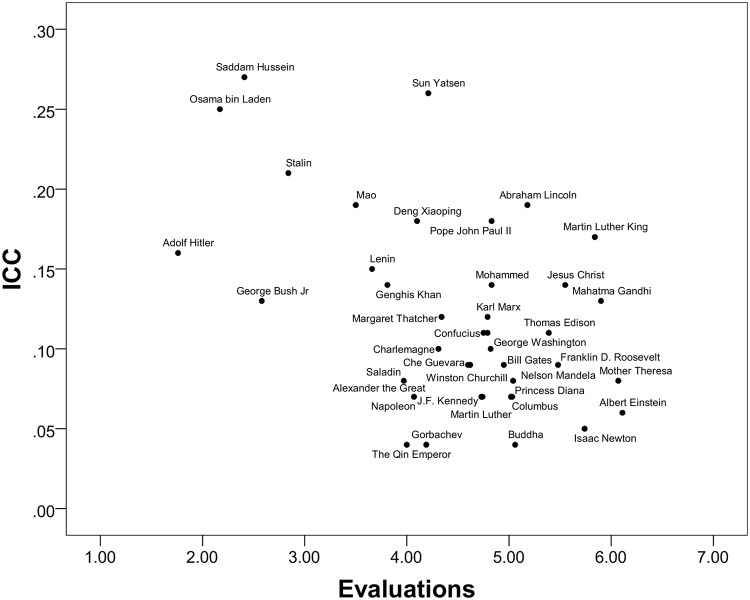
Intraclass correlation coefficients (ICC) and Evaluations scatterplot.

Among the top 10 most admired figures in world history, there was more consensus across countries about Buddha, Newton, Einstein, Mother Theresa and Mandela; in descending order, there was less consensus about Edison, Gandhi, Christ, Martin Luther King, and the least consensus about Lincoln. The pattern suggests that scientists are most consensually evaluated as “heroes” across cultures, together with religious and humanitarian figures who are unambiguously associated with peace. At the bottom, there was less consensus about the evaluation of historical figures, with Saddam Hussein having the highest ICC of any figure, and Osama bin Laden, Stalin, and Mao having the 3^rd^, 4^th^, and 5^th^ highest ICCs in the inventory. The average ICC for the bottom eight (the only real “villains” in terms of mean scores) was. 18 compared to. 10 for the top eight. There was thus more agreement across cultures about the “greatest heroes” of world history compared to its “worst villains”. In Fig. 1, the distribution of the ICCs and the evaluations are plotted. In the upper left quadrant where Saddam Hussein and Osama bin Laden are located was the most variability between the countries moving to the lower right quadrant where there was more similarity between the countries.

We attempted next to map the figures into a meaningful two-dimensional space using a multidimensional scaling procedure (MDS) with Proxscal and subsequent generalized procrustes analysis (GPA) [24]. MDS places objects or in this case the evaluated figures that are similar regarding their evaluation closer together in the MDS space. For example, if Stalin and Hitler are both evaluated negatively, they would be closer together in the MDS space. One could also say that the MDS provides a depiction of the relationships between the figures regarding their valence. When the objects are placed into a two-dimensional space, the researcher has to make sense out of the structure and interpret the dimensions in a meaningful way. GPA then rotates the two-dimensional structure to the point where the differences between all countries become the smallest. Although we removed multiple figures in an attempt to increase the fit (or the similarity) between countries, we were unable to improve the fit. We therefore decided to reduce the number of countries by using a standard hierarchical cluster analysis (CA) which aims to discover fairly homogenous groups of cases. CA commonly uses an algorithm starting with each case (= country) as separate clusters and then continuously clusters these cases until only one cluster is left. The procedure does this by progressively placing objects into clusters which contribute the least error variance.The cluster analysis was based on 35 countries, excluding France and Mauritius. A four cluster solution was superior to a 3, 2 or 1 cluster solution, due to lesser increase of the sum of squared errors.

The cluster analysis thus converged on four clusters of countries: a Catholic-Orthodox (or traditionally Christian) cluster (Argentina, Brazil, Bulgaria, Colombia, Hungary, Mexico, Peru, Portugal, Russia, Spain), a mainly Western cluster (Australia, Austria, Belgium, Canada, Fiji, Germany, Italy, Netherlands, New Zealand, Norway, Philippines, Switzerland, UK, USA), a Muslim cluster (Indonesia, Malaysia, Pakistan, Tunisia), and an Asian cluster (China, Hong Kong, India, Japan, Singapore, South Korea, Taiwan).

**Fig 2:**
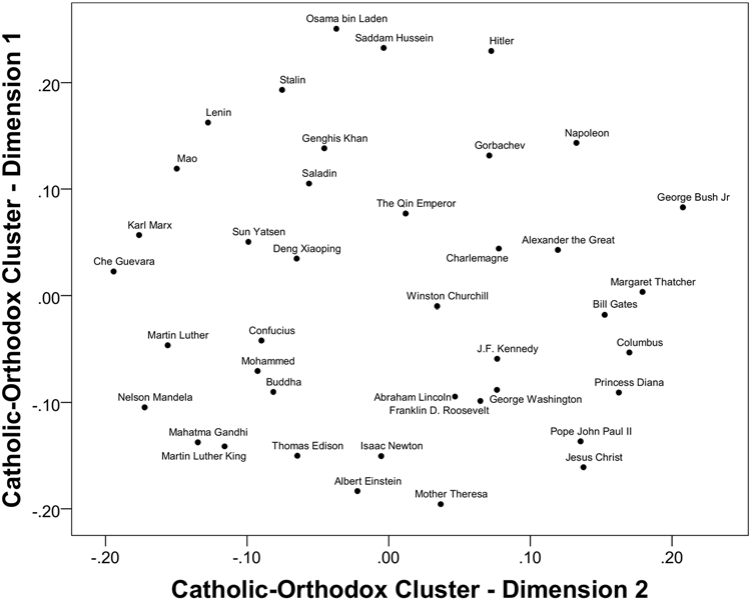
Rotated MDS solution for the Catholic-Orthodox (Argentina, Brazil, Bulgaria, Colombia, Hungary, Mexico, Peru, Portugal, Russia, Spain) cluster with all 40 figures.

**Fig 3:**
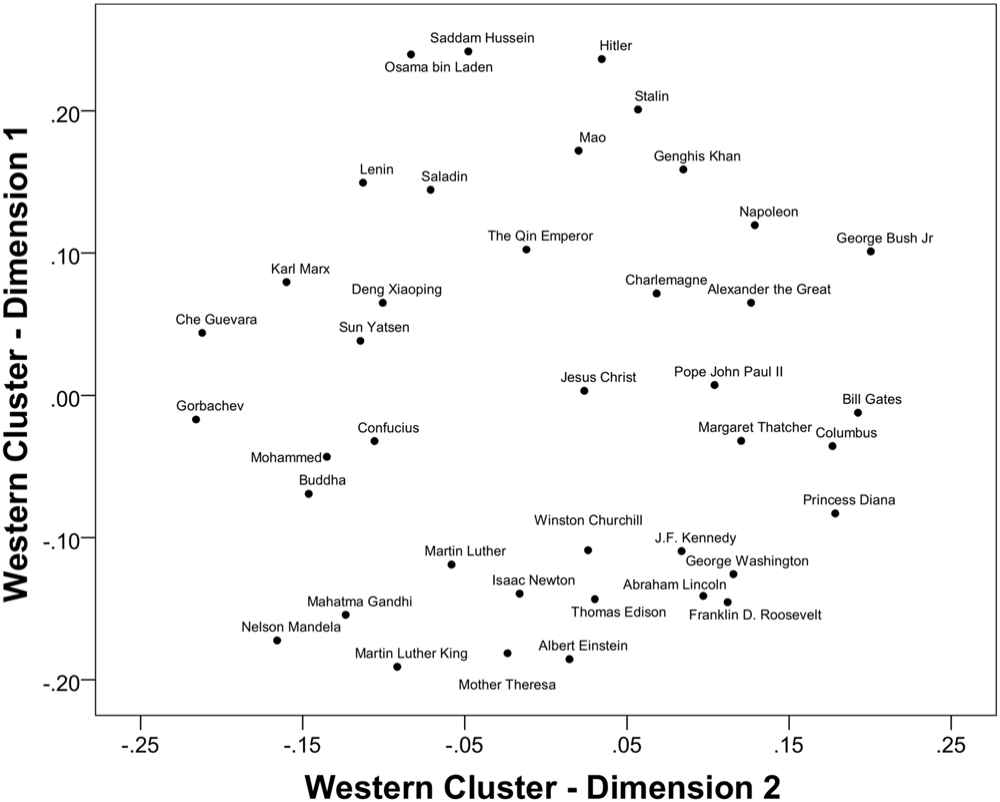
Rotated MDS solution for the Western (Australia, Austria, Belgium, Canada, Fiji, Germany, Italy, Netherlands, New Zealand, Norway, Philippines, Switzerland, UK, USA) cluster with all 40 figures.

Having a reduced set of coherent groups in the form of four country clusters, we repeated the MDS procedure with a subsequent GPA with the following results. The rotated centroid configuration accounted for 69% of the squared distances for two dimensions of evaluations of figures. This was not a satisfactory improvement. The two-dimensional spaces for historical figures were relatively similar only for the first two clusters consisting of predominantly Christian populations. As shown in Fig. 2, the Catholic/Orthodox cluster had two clearly interpretable dimensions: The vertical axis represents positive-negative evaluation, with negative figures at the top and positive figures at the bottom, and the horizontal axis might be termed a "Western Dominance" dimension, with "Western Dominance" to the right and "Resistance to Domination" on the left hand side. The MDS for the Western cluster (see Fig. 3) was similar, suggesting a shared knowledge between the two clusters. The placement of a few of the historical figures varied across the two clusters, with the religious figures Christ and John Paul II having a more positive location for the Catholic/Orthodox cluster than for the Western cluster. The Western cluster may thus apply more secular criteria to evaluations than the Catholic/Orthodox cluster.

**Fig 4:**
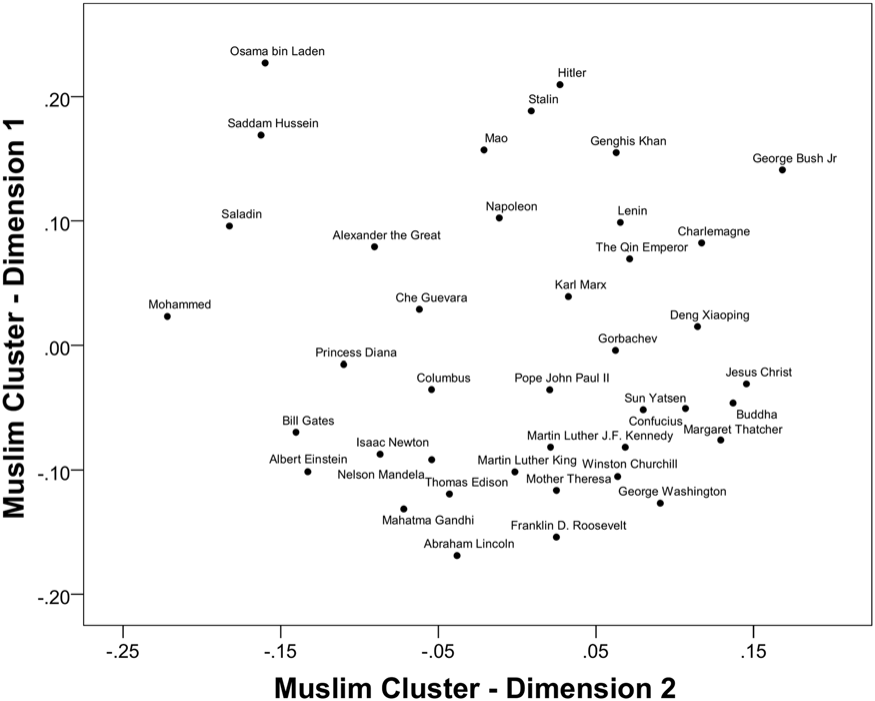
Rotated MDS solution for the Muslim (Indonesia, Malaysia, Pakistan, Tunisia) cluster with all 40 figures.

Interpretation for Fig. 4, the Muslim cluster, is more complex. The vertical axis is still positive/negative, and the general location of most individual figures along this evaluative dimension is reasonably consistent across clusters. Mohammed’s placement in the middle of this dimension is, nonetheless, problematic. On the other hand the horizontal axis is completely distinct: To the left side are all four of the Muslim historical figures, and to the right are a diverse range of others who can only be described as non-Muslim. This suggests an alternative knowledge structure onto which the current selection of 40 figures does not map out very well. More Muslim figures would be needed to uncover the full range of latent meanings behind the ratings of historical figures for Muslim countries.

**Fig 5:**
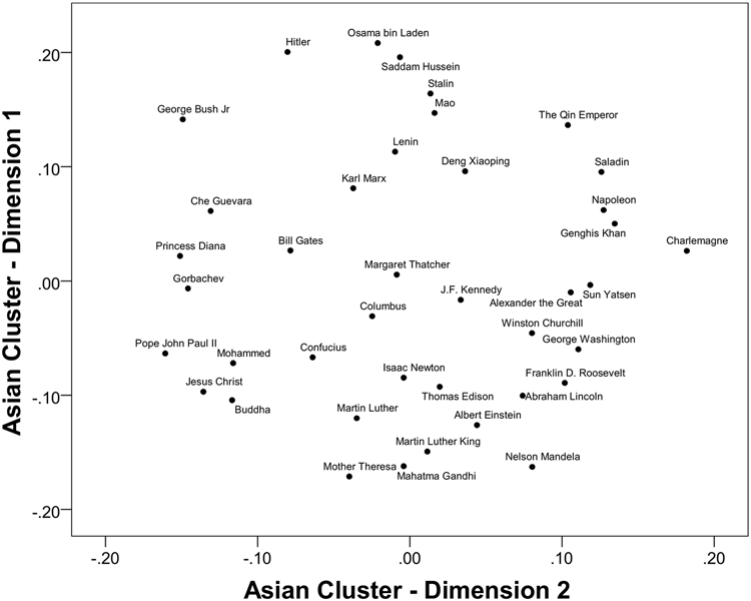
Rotated MDS solution for the Asian (China, Hong Kong, India, Japan, Singapore, South Korea, Taiwan) cluster with all 40 figures.

The horizontal axis of the Asian cluster MDS is not straightforward either, as can be seen in Fig. 5. Pope John Paul II, Princess Diana, George W. Bush, Jesus Christ, and Che Guevara, anchored the left side of the space. The peaceful and democratic Sun Yatsen was on the right side of the MDS anchored by Charlemagne, Genghis Khan, Napoleon, and Saladin. One potential interpretation of the horizontal axis is that it captures diverse aspects of history. More recent figures are on the left and ancient figures on the right. With the exception of the religious figures that could be regarded outside history in many respects due to their continuity as salient figures in people’s lives. This is, however, a highly speculative interpretation. The vertical axis could be interpreted as a positive-negative dimension where negatively evaluated historical figure were anchored on the top and positively evaluated figures at the bottom. It is possible that the Asian cluster may be aggregating across too much variability by combining data from communist China with democratic India (but a 5 or 6 cluster solution did not resolve this issue). The issue that some figures may not be known in some countries adds to a more complex and diverse representational space of historical figures here.

Since our attempts to find universal dimensions of meaning somewhat failed, we decided to introduce a new type of statistical analysis that to our knowledge has not heretofore been employed in dealing with large scale cross-cultural data. It does not presume country as a unit of analysis but rather *constructs* groups of individuals using an inductive procedure that analyzes the configuration of their responses.

### Latent Profile Analysis (LPA)

In the next step, we employed a LPA for the evaluation of 28 historical figures using Mplus [41]. We used LPA to explore how a set of unobserved subgroups of participants may differ reliably in their views of a topic. The set of unobserved subgroups then represents a categorical latent variable (that is a set of distinct categories or types of people) that we hypothesized were producing the overall pattern observed in our data [37]. LPA thus allowed us to create a model categorizing people into different subtypes or categories that are theorized to underlie the overall pattern of responses. LPA has been used to identify different categories or types of people in a range of different research areas: the majority of this work has been in epidemiology where LPA has been used to model disease prevalence [42]. LPA has also been used in various areas of social psychology [37], including research assessing the proportions of people who hold different combinations of religious beliefs [43] and different combinations of sexism [44].

LPA is suited for identifying distinct groups or types of people who may have quite a different set of beliefs or evaluations of different sets of historical events or figures in world history from other distinct types or groups of people. These different types may be overlooked when examining the overall mean levels of responses to a set of attitude or opinion items. Our analysis differentiates people in our data set into latent profiles based on similarities and differences in their overall pattern of responses across a range of continuous indicators—as noted previously, different groups appear to rate controversial figures like Stalin or Mao very differently, and it cannot be assumed that country or culture is the primary determinant of these variances [18].

We examined a range of different solutions, with models ranging from one to five latent profiles. We examined the Sample-size adjusted Bayesian Information Criterion (SABIC) and entropy as indicators of model fit. The SABIC can be used to compare a series of models that differ in the number of parameters. Entropy values range from 0 to 1.0, where a high value indicates a lower classification error. Entropy values close to 1.0 (and typically above. 80) indicate that there is a clear separation of classes, or in other words that the model clearly separates the data into distinct profiles. SABIC and entropy statistics for the different solutions were as follows: single-class solution SABIC = 649585.54; two-class solution SABIC = 638239.86, Entropy = .822; three-class solution SABIC = 631105.97, Entropy = .870; four-class solution (preferred) SABIC = 627066.55, Entropy = .823; five-class solution SABIC = 624197.31, Entropy = .814.

The SABIC and entropy statistics indicated that a four profile solution provided a reasonably parsimonious model of the latent profiles (or discrete categories of people) underlying the observed data. This was further supported by inspecting each solution qualitatively, which indicated that the fifth and subsequent solutions simply provided incrementally finer splits in the profiles identified in the four-class solution without showing any distinctly new patterns (i.e., splitting one class into two profiles that followed the same pattern across all items, but with one class a little higher in terms of mean evaluations than the other). Likewise, a three-class solution did not provide a fine-grained enough solution and failed to differentiate between the religious and secular idealist profiles identified in the four-class solution.

**Fig 6:**
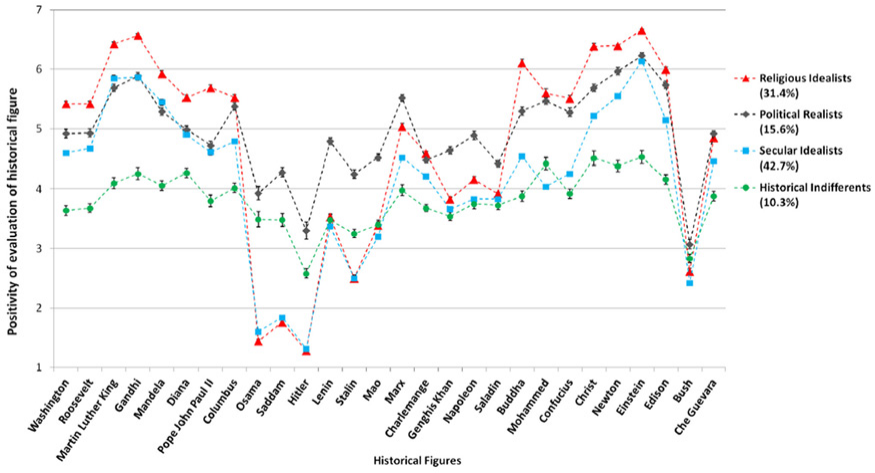
Estimated means for a four-profile Latent Profile Analysis evaluation of historical figures, ordered from left to right according to positions in factor structures (American Presidents, Humanitarians, Explorers, Tyrants, Communists, Conquerors, Religious, Scientists, and Other).

The estimated mean level of evaluation of each historical figure for each of the four *representational profiles* [45] is presented in Fig. 6. After considerable discussion among the authors, we labeled these historical representational profiles “Secular Idealists” (42.7% of the total sample), and “Religious Idealists” (31.4%): together they are termed “Historical Idealists”. Between them may be communicated dominant features of Western discourses concerning historical figures, as a dialogue between secular and (Christian) religious ideas and values. They are contrasted with “Political Realists” (15.6% of the total sample), and “Historical Indifferents” (10.3%). The names chosen for these profiles are highly interpretive. The co-authors on this paper debated these names for two months before settling on the current nomenclature. We caution that these percentages reflect an over-abundance of samples from Western countries (especially from Europe), and in no way mirror the overall population of the world.

Each of these groups appears to represent latent profiles of people that configure the evaluation of historical figures in different but meaningful ways. “Historical Indifferents” were the smallest group in terms of both percentages (10.3%) and showed the least propensity to evaluate historical figures in a manner that reflects deeper meaning: In this representational profile, all figures except Hitler and Bush were rated between 3 and 5, many near the (unlabeled) scale midpoint of 4. This class viewed historical figures with indifference (and lack of knowledge most probably) rather than seeing them as “sinners” or “saints”. On the other hand, the group with the most extreme evaluations were “Religious Idealists”, the second largest class (31.4%): They rated great “Scientists” and “Humanitarians” exceptionally high, “Religious Founders” very highly, were fond of “American Presidents”, and rated “Anti-Heroes” and “Communist Dictators” (excluding Marx himself) extremely negatively. The pattern of their ratings is similar to the largest class, “Secular Idealists” (42.7%) who differed from “Religious Idealists” in that they were more moderate in their evaluations overall, particularly in rating religious figures. In these two profiles, “Conquerors” were rated close to the midpoint. The different categories (“Scientists”, “Humanitarians”, and so forth) are based on a factor analysis which is not reported here due to space restrictions and can be obtained from the authors on request.

These two profiles differed sharply from the fourth class, termed “Political Realists” (15.6%). In this profile, “Anti-Heroes” were rated moderately (close to midpoint), “Communist Dictators” and “Conquerors” were rated somewhat positively, and Karl Marx was rated highly. It is tempting to refer to this class as “Machiavellians”, but in the end we opted for the more evaluatively neutral term. Rather than being purely “Machiavellian”, the “Political Realists” were in line with the “Historical Idealists” in rating “Scientists” and “Humanitarians” highly, and they favored the more theoretical idealists Marx and Lenin over “Communist Dictators” who held power and influence for longer periods of time. They rated “Religious Founders” intermediate between the two profiles of “Idealists”, showing more admiration for religious authority than the “Secular Idealists”.

**Fig 7:**
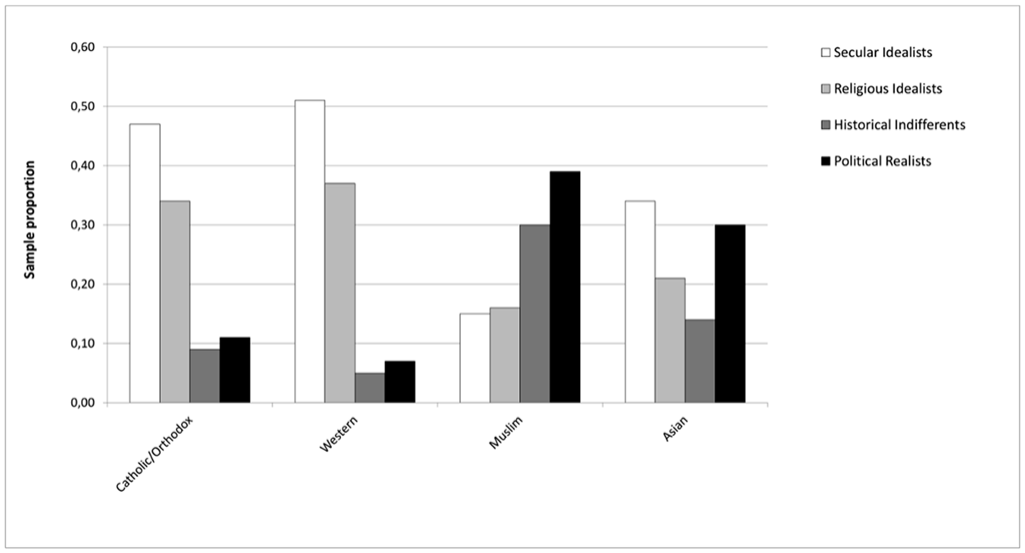
Distribution of historical representation profiles across cultural zones.

We next examined the patterning of cultural differences in each of the four historical representational profiles. We refer to this as *prevalence mapping*, in that we sought to map the prevalence of the four representational profiles in different countries. As can be seen in Fig. 7 (with country details available in Table 2), there were clear differences in the distribution of profiles across regions and countries within regions. Countries in the Western and Catholic/Orthodox clusters tended to contain “Secular” and “Religious Idealists”. These two clusters were similar in the distribution of prevalence mapping, as they were similar in terms of multidimensional scaling. The Western cultural zone was the most homogenous, consisting almost entirely (88%) of “Historical Idealists” (the summed proportion of “Secular Idealists” and “Religious Idealists”). This was compared to 81% “Historical Idealists” in the Catholic/Orthodox country cluster. These numbers reflect the overwhelming influence of Western liberal democratic ideals in “grasping together” [44] or interpreting [46] history among university-educated circles in these predominantly Christian countries. The dialogue between religion and secularity in these countries continues to constitute a source of dialogical and representational dynamism.

**Table 2:** Proportion of sample classified as belonging to each representational profile by nation and by cultural cluster.

Cultural Cluster	Nation	Secular Idealists	Religious Idealists	Historical Indifferents	Political Realists	Sample Size (N)
Catholic/Orthodox	Argentina	.62	.23	.07	.08	346
	Brazil	.43	.34	.10	.13	209
	Bulgaria	.42	.47	.04	.07	237
	Colombia	.39	.31	.12	.18	159
	Hungary	.48	.43	.06	.02	184
	Mexico	.31	.31	.08	.29	198
	Peru	.49	.40	.04	.08	78
	Portugal	.45	.46	.05	.04	189
	Russia	.33	.22	.28	.17	214
	Spain	.63	.29	.05	.02	231
Western	Australia	.57	.31	.09	.03	179
	Austria	.49	.45	.04	.02	194
	Belgium	.73	.24	.01	.02	136
	Canada	.48	.45	.04	.04	195
	Fiji	.31	.42	.09	.18	165
	Germany	.75	.19	.05	.01	146
	Italy	.41	.54	.02	.03	141
	Netherlands	.70	.27	.01	.02	199
	New Zealand	.53	.37	.03	.07	150
	Norway	.74	.23	.02	.01	177
	Philippines	.25	.46	.06	.22	330
	Switzerland	.74	.20	.04	.02	142
	UK	.49	.30	.13	.08	118
	USA	.37	.62	.01	.00	253
Muslim	Indonesia	.17	.25	.24	.33	198
	Malaysia	.13	.14	.44	.30	197
	Pakistan	.15	.09	.31	.44	106
	Tunisia	.16	.08	.11	.66	93
Asian	China	.05	.05	.07	.82	182
	Hong Kong	.19	.11	.34	.36	151
	India	.20	.26	.22	.34	200
	Japan	.53	.18	.16	.13	111
	Singapore	.57	.24	.09	.10	220
	South Korea	.39	.19	.12	.30	217
	Taiwan	.42	.36	.08	.15	284

Countries in the Muslim cluster, by contrast, tended to contain more “Political Realists” (39%) and “Historical Indifferents” (30%). Countries in the Asian zone displayed complex patterns that are harder to summarize, because of the heterogeneity of countries in this zone. Overall there was a high proportion of “Political Realists” (30%) and a similarly high proportion of “Secular Idealists” (34%) but fewer “Religious Idealists” than in the Western or Catholic/Orthodox clusters (21%) (see Fig. 7). The Chinese sample consisted almost entirely of “Political Realists”, whereas the proportions of the four clusters were almost equal in the Indian sample. Singapore, on the other hand, looked a lot like Western countries in its prevalence map (see Table 2).

## Discussion

Several key insights were obtained through analysis of the World History Survey (WHS) examining evaluations of 40 major historical figures from 6,902 students across 37 countries. We are now in a position to make empirically verifiable statements about what global political culture might look like through the lens of young educated adults’ views of historical figures.

### Cross-Cultural Differences and Similarities

First and foremost, we found limited evidence for universality in evaluations of significant figures in world history. Even at the level of regional clusters, our sample of Asian countries produced a multidimensional scaling space for evaluations of historical figures that were not easily interpretable, and the Muslim cluster produced a dimensional space that in no way resembled that for the Western and Catholic-Orthodox clusters. However, there were several specific findings that indicated some commonality in representations of history across countries.

First, there was a high level of global consensus around the virtue of scientific figures: Although all the great scientist figures in the WHS were Westerners, attitudes towards them seem to have transcended in-group favoring or out-group denigrating cultural lenses. Einstein was the most highly rated figure in world history, Newton was 5^th^, and there was little variability in their ratings across cultures. In terms of global political culture, the first salient aspect of it, at least among university students, is that the pursuit of scientific excellence appears to be evaluated in a pan-cultural manner: that is, positively across all sampled cultures. Further research should explore whether this finding holds in non-tertiary-educated samples, where enlightenment values may play a less decisive role.

Following closely behind science, there were a number of humanitarian and religious figures dedicated to championing human rights, relieving human suffering, and providing non-violent resistance to imperialism, colonialism, and racism who were also very highly regarded. Here there was less consensus than for the scientific figures. Mother Theresa was the second most positively evaluated figure in world history, whereas the other peaceful humanitarians Gandhi and Martin Luther King had slightly lower ratings and more cross-cultural variability. Race and gender were no barriers to favorable evaluations. Two of the 10 highest rated figures were Black and two were Asian (Indians); thus four of the 10 most highly rated figures in world history were non-White (about the same ratio of non-Whites in the WHS as a whole, 15 of 40). Two of the top 11 were women.

Association with warfare exacted a penalty on evaluations: among war leaders, only Lincoln was among the top 10 most positively evaluated figures. These student samples appeared to imbue war leaders in history with a substantial degree of agency in pursuing warfare (e.g., holding them responsible by evaluating them lower) in a manner that could interpreted as in accord with Sahlins’ [28] view that individual characters tend to come to the fore as historical actors when something momentous and fraught occurs.

Second, the impact of time could clearly be seen. For example, the very recent figure of George W. Bush was rated as more negative than Joseph Stalin, who was responsible for innumerably more deaths. The effects of temporal distance can also be seen from the almost neutral rating received by Genghis Khan, which is not the result one might have expected given the amount of human suffering and cultural destruction he unleashed on the 13^th^ century. Such mid-point ratings were also given to Saladin and the Qin Emperor, also ancient conquerors and empire builders, and to Napoleon, who is not as ancient, but considerably less controversial today than he would have been in the 19^th^ century. Time seems to push these historical conquerors towards neutral evaluations as living memories of them fade [47], [48] or are simply not that well-known.

Third, and perhaps most importantly respondents regarded figures associated with the rise of human rights and resistance to oppression with a relatively high degree of positive consensus. That is, there was more consensus about the positive valence of particular historical figures compared to the historical figures evaluated as negative in world history. While the genocidal Hitler was undisputed as the worst rated figure in world history, his ICC (showing cross-cultural disagreement) was much higher than that for the benevolent figures of Albert Einstein and Mother Theresa. Even more controversy surrounded Osama bin Laden and Saddam Hussein, who were rated close to the midpoint in our small (n = 4 countries) and non-representative (mostly Asian countries) sample of Muslim nations but negatively everywhere else.

Our findings thus seem to suggest that where evaluations of negative figures in particular are concerned, there may be highly in-group favoring discourses that provide means-ends justifications for rating figures like Mao, Stalin, and Lenin favorably and Osama and Saddam towards the midpoint. That is, according to ingroup accounts, Mao, Stalin and Lenin may have “made mistakes”, but they also made hard choices required for nation-building, whereas Osama and Saddam may be viewed as “fighters against Western imperialism”. We suspect that these means-ends justifications of less favored figures are culture-specific, and may counter the homogenization of global political culture among young educated people even though there is substantial agreement about its “heroes” and preferred values. From our MDS analyses, it appears that some Asian and Muslim countries may have alternative systems for mapping the meaning of figures in world history, supporting Sahlins’ [28] contention that culture provides the means to make sense of history. Unfortunately, we did not have enough Muslim and Asian figures to fully map out this meaning space; there is also some doubt as to whether such figures would be coherent across the whole spectrum of different Muslim and Asian countries. The Western and Catholic-Orthodox clusters that share civilizational roots as predominantly Christian countries appeared to also share a similar view of world history as emanating from positive advances from the West and reactions to them [48]. But of course, the generality of these findings is limited by our university educated samples, and the fact that the availability and affordability of tertiary education varies widely across cultures. Furthermore, a more comprehensive sampling of countries would add to the confidence of the cultural zone cluster classifications that we have produced.

### Representational Profiles

Our analyses of representational profiles revealed a gap between Western and Catholic/Orthodox clusters versus Muslim and Asian countries clusters in prevalence mappings. The Western clusters and Catholic/Orthodox clusters consisted almost entirely (88%) of “Historical Idealists”, whereas the Muslim cluster had a plurality of “Political Realists” (39%) and “Historical Indifferents” (30%). The “Historical Indifference” found in Muslim countries may be a product of a rejection of Eurocentrism in the selection of historical figures (including relatively few Muslims). The Asian country cluster was more of a hybrid, containing a plurality of “Secular Idealists” (34%) and “Political Realists” (30%).

This pattern of cross-cultural similarities and differences is useful for understanding the roots, branches, and future trajectories of nascent global political culture. First, the similarities between Western countries and Catholic (mainly Latin American) and Orthodox (mainly post-Communist) countries in our sample suggest that university-educated young people in these countries have *ensemble* representations of historical figures strongly influenced by Western liberal democratic ideals. History is a crucial symbolic resource [4] for legitimizing political systems, and our findings suggest a dialogical process by which governments are called upon to live up to their often unfulfilled ideals by exceptional figures, like the Gandhis and Martin Luther Kings of the world, who are then valorized subsequently by large numbers of their educated citizens (who might have opposed their liberalizing agendas during the great humanitarians’ lifetimes). We could argue that an *End of History* in Fukuyama’s terms [20] is indeed taking place, where the most enduring form of social order is liberal democracy. However, it is clear that even this “end” at times will be characterized by a fierce debate between “Secular” and “Religious Idealists” in Western and Catholic/Orthodox countries. Cosmopolitan theorists such as Nussbaum [13] have had to contend with criticism that their formulations of the global citizen are too much based on Kantian ideals of rationality, and not sufficiently grounded in more intuitive feelings [49], and less tolerant identities such as religious ones [14], [21].

Our four Muslim countries provide a powerful counterpoint against over-generalizing this pattern, made all the more poignant by the fact that one of the four samples was Tunisia, the tinderbox that started the so-called Arab Spring. In a global representational framework, it is incumbent upon the citizens of Western countries to understand that the overwhelming support for “Historical Idealism” prevalent in their countries is not universal, and cannot be expected to be transplanted without growing pains to Muslim countries. Rather, we should anticipate a long trajectory of democratization in these countries rather than an unbroken chain of immediate successes, as localized and marginalized formations are very important even in the analysis of cosmopolitanism [50]. Because we only included four Muslim figures in the World History Survey, and because differences between university and non-university educated are likely to be important, there is much research to be done on representations of history in Muslim countries.

Asia contained such a diversity of attitudes and beliefs about history that the MDS for the region was very complex: it is organized as a geographical rather than as an attitudinal whole in our data [51]. How can a single representation accommodate both India’s democracy and the Market Leninism of China? In this region, aggregating across seven countries, there was both a high prevalence of “Secular Idealists” (but not “Religious Idealists”) and “Political Realists”, showing respect for Western icons of liberal economics *and* a respect for the top-down, authoritarian traditions that governed Asia prior to recent times in many places [11]. This prevalence mapping shows signs of a fully-engaged dialogue between Western traditions of historical interpretation, rooted in liberal ideals of free trade, private property, democracy, and human rights on the one hand, and Asian traditions, rooted in sovereign wealth, collectivist schemes of ownership, autocratic governance, and ethics of hierarchical relationalism on the other [49], [50]. The heterogeneity of these representations co-occurs with the construction of hybrid forms of governance from the family-based democratic oligarchies in the Philippines to “Socialism with Chinese characteristics” in China and the one-party democracies of Singapore (and late 20^th^ century Japan). Such diversity of solutions to issues of the authority of leaders and governance is an important legacy of history to global political culture that cannot and should not be swept away under homogenizing discourses involving modernity or globalization [52].

## Conclusion

We are at a juncture in time where the world is becoming fully connected economically [1]. Understanding the structure and evaluation of global ratings of historical figures allows an understanding of what emergent global political culture might look like as a parallel and culturally distributed system. The core of the “big picture” identified in this research is one that acknowledges the dominant influence of the “Historical Idealism” rooted in Western civilization, but is fully inclusive of the diversity of global political culture that exists in large parts of the Islamic world and Asia. Our method of ascertaining representational profiles underlines that different positions across countries are not restricted by a priori factors like nationality or religion, but are acknowledged as alternative branches of global political culture that are marked by different historical trajectories emerging out of different cultural origins.
